# Supplementary material for: Evaluating the comprehensive water resources utilization level in China: Dynamic distribution analysis and spatial convergence insights
Source: PLoS One. 2025 Mar 3;20(3):e0319150. doi: 10.1371/journal.pone.0319150 (PMC11875361; doi:10.1371/journal.pone.0319150)
Supplement: Attachment — Submitted filename: Review Comments for PLOSONE.pdf [file pone.0319150.s002.pdf]

I have read the manuscript titled "Evaluating the Comprehensive Water Resources Utilization Level in China: Dynamic Distribution Analysis and Spatial Convergence Insights". The manuscript can be accepted for publication only after the authors agree to include **major revisions.** Here are some comments and observations on the manuscript:

1. Ensure the authors' affiliations are correctly formatted
2. The abstract is comprehensive but could benefit from specific quantitative results or metrics to provide more concrete insights into the findings.
3. The document shows inconsistencies in the formatting of titles, section headings, and paragraph spacing. For instance, "2. Research design" and "1.Introduction" display inconsistencies in numbering and punctuation.
4. The introduction section can be further strengthened by providing more background information on the existing challenges in water resource management globally to set a broader context.
5. Certain phrases and concepts are repeated unnecessarily. For example, the abstract and introduction both contain repetitive details regarding regional disparities and policy impacts.
6. Ensure the consistent use of terms and acronyms throughout the manuscript, such as "CWRULE," to maintain clarity for the reader.
7. Elaborate on the sources of the data used in the study and discuss any potential limitations or biases in the data. This will provide context and strengthen the validity of the findings.
8. Include more details on the methodological framework, especially the rationale for choosing the Dagum Gini coefficient, spatial kernel density estimation, and spatial convergence models. Explain why these methods are suitable for the study.
9. Ensure that all figures and tables are adequately labelled, captioned, and referenced in the text. Table name and number should be in the same line. Don’t start a sentence by writing “Fig.4 displays changes in the……” Or end the sentence with “……..the calculated results are presented in Fig.6”. Instead cite the table or the figure within the text after a sentence by using brackets.
10. Expand the discussion to include comparisons with similar studies or contrasting results. Discuss the limitations of the study and propose how future research can address these gaps.
11. Discuss the implications of the findings for policymakers, especially concerning the identified regional disparities. Suggest specific policy measures that could address the issues highlighted by the study.
12. Strengthen the conclusion by summarizing the key findings and emphasizing their importance. Also, suggest areas for future research that could build on the current study's findings.

Implementing these corrections and suggestions will likely enhance the readability and impact of the manuscript, making it more accessible and informative to the audience.
