# Supplementary material for: Evaluating the comprehensive water resources utilization level in China: Dynamic distribution analysis and spatial convergence insights
Source: PLoS One. 2025 Mar 3;20(3):e0319150. doi: 10.1371/journal.pone.0319150 (PMC11875361; doi:10.1371/journal.pone.0319150)
Supplement: Attachment — Submitted filename: Review-report.docx [file pone.0319150.s001.docx]

**Abstract:**

- The abstract lacks an introductory statement and conclusion. Begin the abstract with a brief introduction to the topic, followed by the main objectives, key findings, and a concluding sentence summarizing the implications of the research.
- Keywords are too long. Use shorter and more specific keywords that are directly relevant to the study.

**Introduction:**

- The introduction section needs to incorporate recent empirical literature from around the world related to the topic. Review and include recent studies that discuss water resource utilization and spatial convergence to provide a comprehensive background.
- Research gaps are not clearly identified. Clearly articulate the existing research gaps that this study aims to address.
- Objectives are missing from the end of the introduction. List the specific objectives of the study at the end of the introduction section to provide a clear roadmap for the reader.

**Methodology:**

- The methodology section is good but lacks organization (i.e., Data collection, processing, and analysis). Clearly separate the methodology into subsections such as Data Collection, Data Processing, and Data Analysis for better organization.
- Mixing of methodological flow chart with the conceptual framework. Create a separate methodological flow chart to visualize the steps taken in the study, and keep the conceptual framework distinct.

**Results:**

- All maps need to be regenerated with a higher DPI of 1000 resolution to make the maps and legends clear. Ensure all maps are of high resolution to improve readability and clarity.
- Titles for each line graph on Figure 4 are not needed. Use (a), (b), etc., on the graphs and provide a proper caption for each letter, avoiding individual titles for clarity.

**Discussion:**

The discussion section is missing from the manuscript. Add a discussion section that interprets the results, compares them with existing literature, and discusses the implications and limitations of the findings.

**Conclusion:**

- The conclusion is unclear and does not summarize the major findings. Write a clear conclusion that summarizes the major findings of the study in relation to the research questions or hypotheses. Highlight the implications and possible future research directions.

**Overall Recommendation:**

The manuscript is scientifically sound and addresses an important issue. However, significant revisions are needed to improve clarity, organization, and completeness. Incorporating these changes will enhance the manuscript's quality and readability, making it suitable for publication.
