# Supplementary material for: Evaluating the comprehensive water resources utilization level in China: Dynamic distribution analysis and spatial convergence insights
Source: PLoS One. 2025 Mar 3;20(3):e0319150. doi: 10.1371/journal.pone.0319150 (PMC11875361; doi:10.1371/journal.pone.0319150)
Supplement: Attachment — Submitted filename: Response to Reviewers.docx [file pone.0319150.s003.docx]

**Response to Reviewers**

Response to Reviewer #1:

**Thank you for the reviewer’s comments. In response to your suggestions, we have made the following revisions:**

**Abstract:**

• The abstract lacks an introductory statement and conclusion. Begin the abstract with a brief introduction to the topic, followed by the main objectives, key findings, and a concluding sentence summarizing the implications of the research.

• Keywords are too long. Use shorter and more specific keywords that are directly relevant to the study.

Thank you for pointing this out. We have revised the abstract by adding an introductory statement that briefly introduces the topic of water resources utilization in China. We have also included a concluding sentence that summarizes the key implications of the research. The keywords have been shortened and refined to be more specific and directly relevant to the study.

**Introduction:**

• The introduction section needs to incorporate recent empirical literature from around the world related to the topic. Review and include recent studies that discuss water resource utilization and spatial convergence to provide a comprehensive background.

• Research gaps are not clearly identified. Clearly articulate the existing research gaps that this study aims to address.

• Objectives are missing from the end of the introduction. List the specific objectives of the study at the end of the introduction section to provide a clear roadmap for the reader.

We appreciate your suggestion. We have revised the introduction to include more recent empirical studies on water resource utilization and spatial convergence from around the world. The research gaps are now clearly identified, and we have explicitly stated the objectives at the end of the introduction to provide a clear roadmap for the reader.

**Methodology:**

• The methodology section is good but lacks organization (i.e., Data collection, processing, and analysis). Clearly separate the methodology into subsections such as Data Collection, Data Processing, and Data Analysis for better organization.

• Mixing of methodological flow chart with the conceptual framework. Create a separate methodological flow chart to visualize the steps taken in the study, and keep the conceptual framework distinct.

Thank you for the feedback. We have reorganized the methodology section into distinct subsections: Data Collection, Data Processing, and Data Analysis. Additionally, we have separated the methodological flow chart from the conceptual framework, making them clearer and easier to follow.

**Results:**

• All maps need to be regenerated with a higher DPI of 1000 resolution to make the maps and legends clear. Ensure all maps are of high resolution to improve readability and clarity.

• Titles for each line graph on Figure 4 are not needed. Use (a), (b), etc., on the graphs and provide a proper caption for each letter, avoiding individual titles for clarity.

Due to copyright issues related to the maps, we have decided to remove all the relevant maps and will no longer regenerate them. Additionally, Figure 4 has been revised according to your suggestion: the individual titles have been removed, and the line graphs have been labeled with (a), (b), etc., with proper captions provided for each section.

**Discussion:** The discussion section is missing from the manuscript. Add a discussion section that interprets the results, compares them with existing literature, and discusses the implications and limitations of the findings.

We apologize for this oversight. A comprehensive discussion section has now been added. It interprets the results, compares them with existing literature, and discusses the implications and limitations of the findings.

**Conclusion:** The conclusion is unclear and does not summarize the major findings. Write a clear conclusion that summarizes the major findings of the study in relation to the research questions or hypotheses. Highlight the implications and possible future research directions.

We have revised the conclusion to clearly summarize the major findings of the study in relation to the research questions. Additionally, we have highlighted the implications and potential directions for future research.

**We are confident that these revisions have greatly enhanced the clarity, organization, and overall quality of the manuscript. Once again, we sincerely appreciate your thoughtful and detailed feedback, which has been instrumental in improving the rigor and presentation of our research.**

Response to Reviewer #2: I have read the manuscript titled "Evaluating the Comprehensive Water Resources Utilization Level in China: Dynamic Distribution Analysis and Spatial Convergence Insights". The manuscript can be accepted for publication only after the authors agree to include major revisions.

**Thank you for the reviewer’s insightful comments and suggestions. We have carefully considered each point and made the following revisions to address the concerns:**

1. Ensure the authors' affiliations are correctly formatted

We have reviewed and corrected the formatting of all authors' affiliations to ensure they are in accordance with the journal’s guidelines.

2. The abstract is comprehensive but could benefit from specific quantitative results or metrics to provide more concrete insights into the findings.

We have included specific quantitative results in the abstract to provide clearer insights into the key findings and make the summary more concrete.

3. The document shows inconsistencies in the formatting of titles, section headings, and paragraph spacing. For instance, "2．Research design" and "1.Introduction" display inconsistencies in numbering and punctuation.

We have addressed the formatting inconsistencies, including the numbering and punctuation in section titles such as “Research design” and “Introduction.” These are now aligned with the journal’s formatting requirements.

4. The introduction section can be further strengthened by providing more background information on the existing challenges in water resource management globally to set a broader context.

The introduction has been expanded to include a broader context, highlighting global challenges in water resource management, which sets a stronger foundation for the study.

5. Certain phrases and concepts are repeated unnecessarily. For example, the abstract and introduction both contain repetitive details regarding regional disparities and policy impacts.

We have revised the abstract and introduction to eliminate any unnecessary repetition, particularly regarding regional disparities and policy impacts, ensuring the content is concise and focused.

6. Ensure the consistent use of terms and acronyms throughout the manuscript, such as "CWRULE," to maintain clarity for the reader.

We have carefully reviewed the manuscript to ensure consistent use of terms and acronyms such as "CWRULE," maintaining clarity and avoiding any confusion for the reader.

7. Elaborate on the sources of the data used in the study and discuss any potential limitations or biases in the data. This will provide context and strengthen the validity of the findings.

We have provided additional details on the data sources used in the study and have included a discussion on any potential limitations or biases that may affect the findings, thereby strengthening the validity of the research.

8. Include more details on the methodological framework, especially the rationale for choosing the Dagum Gini coefficient, spatial kernel density estimation, and spatial convergence models. Explain why these methods are suitable for the study.

The methodological framework has been expanded to explain the rationale behind the selection of methods such as the Dagum Gini coefficient, spatial kernel density estimation, and spatial convergence models. This helps clarify why these methods are appropriate for the study.

9. Ensure that all figures and tables are adequately labelled, captioned, and referenced in the text. Table name and number should be in the same line. Don’t start a sentence by writing “Fig.4 displays changes in the…….” Or end the sentence with “……..the calculated results are presented in Fig.6”. Instead cite the table or the figure within the text after a sentence by using brackets.

We have reviewed all figures and tables to ensure they are properly labelled, captioned, and referenced in the text. We have also followed the recommendation to cite figures and tables within the sentence rather than starting or ending with them.

10. Expand the discussion to include comparisons with similar studies or contrasting results. Discuss the limitations of the study and propose how future research can address these gaps.

The discussion has been expanded to include comparisons with similar studies and contrasting findings where relevant. Additionally, we have addressed the limitations of the study and suggested future research directions to address these gaps.

11. Discuss the implications of the findings for policymakers, especially concerning the identified regional disparities. Suggest specific policy measures that could address the issues highlighted by the study.

We have added a section discussing the policy implications of our findings, particularly in relation to regional disparities. We have also suggested specific policy measures that could help address the issues identified in the study.

12. Strengthen the conclusion by summarizing the key findings and emphasizing their importance. Also, suggest areas for future research that could build on the current study's findings.

The conclusion has been strengthened by clearly summarizing the key findings and emphasizing their importance. We have also suggested areas for future research that could build on the current study’s findings.

We believe these revisions have significantly improved the manuscript’s clarity, readability, and overall impact. Thank you once again for your constructive feedback, which has been invaluable in enhancing the quality of our work.
