# Supplementary material for: Evaluating the comprehensive water resources utilization level in China: Dynamic distribution analysis and spatial convergence insights
Source: PLoS One. 2025 Mar 3;20(3):e0319150. doi: 10.1371/journal.pone.0319150 (PMC11875361; doi:10.1371/journal.pone.0319150)
Supplement: Attachment — Submitted filename: Review Comments 2.docx [file pone.0319150.s004.docx]

The authors have addressed several critical revisions based on reviewer feedback, including reworking the abstract, updating keywords, expanding the literature review, reorganizing the methodology, and enhancing the discussion and conclusion sections. Although the paper needs some minor revisions to be incorporated:

1. The convergence analysis results could benefit from a more detailed discussion on why specific regions like the East show weaker convergence. Exploring potential socio-economic or policy-based factors underlying these differences could lead to stronger regional policy recommendations.
2. The policy suggestions are well-developed but could include examples of successful policy models from other regions or countries that address similar issues (e.g., drought management in California). This would provide actionable insights beyond theoretical recommendations.
3. While the conclusion summarizes findings well, it could emphasize the urgency of water management disparities and the need for rapid intervention in underperforming regions to better highlight the study's importance.
