# Supplementary material for: Evaluating the comprehensive water resources utilization level in China: Dynamic distribution analysis and spatial convergence insights
Source: PLoS One. 2025 Mar 3;20(3):e0319150. doi: 10.1371/journal.pone.0319150 (PMC11875361; doi:10.1371/journal.pone.0319150)
Supplement: Attachment — Submitted filename: Response_to_Reviewers_auresp_3.docx [file pone.0319150.s006.docx]

**Response to Reviewers**

Additional Editor Comments:

Please revise the entire manuscript thoroughly, addressing the previous reviewer comments comprehensively. Ensure that all feedback is carefully considered and effectively incorporated. Importantly, it has come to my attention that parts of the manuscript include AI-generated content. If AI tools were used in drafting or editing, this must be explicitly acknowledged in the manuscript. Additionally, ensure that no AI-generated text has been copied and pasted directly without proper cross-checking for accuracy, originality, and relevance. For instance, generic phrases such as “Thank you for considering our revised manuscript for publication in [Journal Name]” appear to reflect standard AI-generated language and should be critically evaluated or rewritten to maintain authenticity and alignment with the manuscript's style. Please prioritize originality and accuracy throughout the revision process.

We have noted the editor's comment regarding the potential inclusion of AI-generated content in the manuscript. We would like to clarify that no AI tools were used in the writing or editing of this manuscript. All text is original, authored by us, and has undergone rigorous review and revision to ensure its accuracy, originality, and relevance. We understand the importance of originality and accuracy, and have thoroughly reviewed the manuscript to ensure that no unverified content is included.

Reviewer #2: The authors have addressed several critical revisions based on reviewer feedback, including reworking the abstract, updating keywords, expanding the literature review, reorganizing the methodology, and enhancing the discussion and conclusion sections. Although the paper needs some minor revisions to be incorporated:

1. The convergence analysis results could benefit from a more detailed discussion on why specific regions like the East show weaker convergence. Exploring potential socio-economic or policy-based factors underlying these differences could lead to stronger regional policy recommendations.

2. The policy suggestions are well-developed but could include examples of successful policy models from other regions or countries that address similar issues (e.g., drought management in California). This would provide actionable insights beyond theoretical recommendations.

3. While the conclusion summarizes findings well, it could emphasize the urgency of water management disparities and the need for rapid intervention in underperforming regions to better highlight the study's importance.

Response to Reviewer 2's First Comment:

1.The convergence analysis results could benefit from a more detailed discussion on why specific regions like the East show weaker convergence. Exploring potential socio-economic or policy-based factors underlying these differences could lead to stronger regional policy recommendations.

We sincerely thank the reviewer for their valuable comments. In response to the issue of weaker convergence in the eastern region, we have conducted a more detailed discussion on the underlying socio-economic and policy factors. Due to the already high baselinethe comprehensive water resources utilization level (CWRULE) in the eastern region, the "law of diminishing marginal returns" applies, meaning that further improvements in CWRULE are limited. Additionally, the highly developed industrialization and urbanization in the eastern region have led to a dominance of water-intensive industries, resulting in continuously high water demand. However, improvements through high-tech investments are often difficult to achieve in the short term. Furthermore, the complex administrative structures and coordination challenges among multiple stakeholders have weakened the effectiveness of policy implementation. Existing policies tend to focus on maintaining high CWRULE rather than supporting innovative breakthroughs. To address this, we have added specific policy recommendations for the eastern region, including the development and promotion of innovative water management technologies such as smart water systems and real-time monitoring tools, the implementation of stricter industry water-saving policies (e.g., tax incentives and water rights trading), and the strengthening of cross-regional coordination and resource optimization. These analyses and recommendations further deepen our understanding of the challenges in the eastern region and provide practical solutions for improving its CWRULE.

Response to Reviewer 2's Second Comment:

2. The policy suggestions are well-developed but could include examples of successful policy models from other regions or countries that address similar issues (e.g., drought management in California). This would provide actionable insights beyond theoretical recommendations.

We sincerely thank the reviewer for their valuable comments. In response to the reviewer’s suggestion to “incorporate successful cases from other regions or countries,” we have added several international policy models and their insights to the policy recommendation section. For example:

(1) California’s drought management: We highlighted its Managed Aquifer Recharge (MAR) programs, which effectively alleviated water shortages during droughts by restoring groundwater reserves. Additionally, California’s “Save Our Water” campaign significantly raised public awareness of water conservation, playing a crucial role in drought management. These experiences offer valuable lessons for addressing water scarcity in China’s western regions.
(2) Israel’s agricultural irrigation technology: Israel has significantly improved agricultural water efficiency through advanced irrigation systems, such as drip and sprinkler irrigation. This provides a useful reference for China’s central and northeastern regions, which are water-rich but have lower efficiency in agricultural water use.

(3)Singapore’s “NEWater” initiative: This program achieves the recycling of wastewater into industrial and potable water, offering inspiration for water-saving policies in China’s water-intensive industrial sectors.

(4)Australia’s basin management experience: The Murray-Darling Basin Authority’s cross-regional coordination mechanism ensures the sustainable allocation of water resources, providing practical insights for China to establish cross-regional water rights management and resource allocation policies.

The incorporation of these international success stories not only enhances the practical applicability of the policy recommendations but also provides diverse solutions to address China’s regional water resource management challenges.

Response to Reviewer 2's Third Comment:

3.While the conclusion summarizes findings well, it could emphasize the urgency of water management disparities and the need for rapid intervention in underperforming regions to better highlight the study's importance.

We sincerely thank the reviewer for their valuable comments. In the conclusion section, based on the reviewer’s suggestion to "emphasize the urgency of addressing water resource management imbalances and the necessity of rapid intervention," we have highlighted the significant disparities in water resource utilization efficiency (CWRULE) across different regions in China. Specifically, the low water resource management efficiency in the western and northeastern regions poses potential threats to regional development and the sustainable utilization of water resources nationwide. The paper further proposes that prompt intervention is needed in underperforming regions, including increased investment in infrastructure, promotion of efficient water-use technologies, and optimization of policy support, to narrow the regional gaps. Additionally, the study emphasizes its significance by not only identifying the disparities in regional water resource management but also providing theoretical foundations for formulating region-specific water resource policies. These revisions make the study’s conclusions more aligned with real-world challenges and underscore its value in guiding policymaking.

By revising the sections on convergence analysis, policy recommendations, and conclusions, the paper further deepens its analysis of the research problem, incorporates international perspectives, and strengthens the practical applicability of the policy recommendations. These adjustments enhance the importance and urgency of the research, making the paper more rigorous and solution-oriented. We sincerely thank the reviewer for their valuable comments, which have greatly contributed to the improvement of our manuscript.

2025.1.28
